# Correlation of soft tissue biotype with pink aesthetic score in single full veneer crown

**DOI:** 10.6026/973206300161139

**Published:** 2020-12-31

**Authors:** Sanjog Agarwal, Subhabrata Maiti, V Ashok

**Affiliations:** 1 Saveetha Dental College, Saveetha Institute of Medical and Technical Sciences, Saveetha University, Chennai 77, India

**Keywords:** Gingival biotype, PES index, single crown, classification

## Abstract

It is of interest to document the correlation of soft tissue biotype with pink esthetic score in single full veneer crown in Indian population. Hence, a Cross-sectional, descriptive study was conducted in an institution, on randomly selected individuals from a data collection of 86000 patient data. Scalloped and thin gingival biotype was present in 62.1 % patients and flat and thick was present in 37.9% individuals according to Anon and Ross Classification. Pink esthetic score didn't give any significant value in single crown cases where 85% cases had a good pink aesthetic score. Thus, the rightness of the PES index for the objective outcome assessment of the esthetic dimension of anterior single-tooth crown was confirmed. However, many randomized clinical trials are needed to further validate and refine this index for its clinical use in prosthetic rehabilitation.

## Background

Understanding the gingival aspect of restorative dentistry is important in harmonizing esthetics and biological function. Dentistry began as a specialty catering to merely the functional needs of patients. Through its evolution, it has come a long way and now is driven primarily by esthetics. In this era of esthetic driven dentistry, it is paramount that clinicians consider how gingiva will respond to the various restorative, prosthetic, and periodontal procedures. Ochsenbein and Ross [1] first indicated that there were two main types of gingival morphology, namely the scalloped and thin or flat and thick gingiva. Seibert and Lindhe to categorize the gingiva into "thick-flat" and "thin-scalloped" biotypes later introduced a more comprehensive term "periodontal biotype". Anon and Ross classified them as thick and thin biotypes. Currently, the term gingival biotype has been used to describe the thickness of the gingiva in the facio-palatal dimension [2 - check with author] [3] Thick gingival tissues are relatively dense in appearance with a rather wide zone keratinized gingiva. On the other hand, a thin biotype is delicate and translucent, friable with a minimum zone of attached gingiva [4,5]. All these studies proposed the existence of two types of gingival biotype, namely thin and thick [6,7]. Gingival morphology identification is considered important because differences in soft and hard tissue architecture have shown to exhibit a significant impact on the final esthetic outcome of restorative therapy, periodontal therapy, root coverage procedures, and implant esthetics [8-11]. Variations in bone and gingival architecture may lead to different tissue responses. In general, patients with a thin biotype are considered to have a higher risk of aesthetic complications after surgical or restorative treatments [9, 10,12-14]. On the other hand, thicker biotypes can originate gingival regrowth and poorer outcomes [11,15, 16]. Probe visibility through gingival sulcus has been strongly associated with clinical classification of thin biotype, while inability to visualize has been associated with clinical classification of thick biotype [17]. There are a total of five variables which are considered before giving a score first being mesial papilla, second distal papilla, third curvature of the facial mucosa, fourth level of the facial mucosa, and last root convexity/soft tissue color and texture at the facial aspect of the site. A score of 2, 1, or 0 is applicable to all five PES parameters. There are two papillary scores [mesial and distal] which are assessed for the complete presence which gives [score 2], incomplete presence which gives [score 1], or absence hence [score 0] of papillary tissue. The curvature of the facial soft tissue line is defined as the line of emergence of the implant restoration from the soft tissues, and is evaluated as being identical to [score 2], slightly different to [score1], or markedly different to [score 0] compared to the natural control tooth and, thus, provides a natural symmetrical or disharmonious appearance. The level of the facial peri-implant mucosa is scored to the contra lateral tooth in terms of an identical vertical level to [score 2], a slight [1mm] discrepancy to [score 1], or a major [1mm] discrepancy [score 0]. Finally, the proposed index combines three additional specific soft tissue parameters as one variable: the presence, partial presence, or absence of a convex profile [in analogy to a root eminence] on the facial aspect, as well as the related mucosal color and surface texture. The latter two elements basically reflect the presence or absence of an inflammatory process, which, in turn, may adversely affect the appearance of an anterior single-tooth restoration. To get a score of 2 for this,all three parameters are more or less identical compared to the control tooth. A value of 1 is assigned if two criteria are fulfilled, whereas a score of 0 is assigned if none or only one parameter matches the control site.The five described parameters are added up in the end add up to a maximum score of 10 if all conditions are ideal (Figure 1 - see PDF). Therefore, it is of interest to document the correlation of soft tissue biotype with pink esthetic score in single full veneer crown in Indian population.

## Material and methods: 

The study setting for this study is a university study setting, which was done on Indian population to study correlation between demographic data and gingival biotype and validity of PES index (Table 1 - see PDF) on single crown restorations. Approval for the study was taken from the ethical board of Saveetha dental college and hospitals [SIMAT]. There were two reviewers involved to examine the results of the study.

The data included in the study was from June 2019-March 2020.26000case sheets were reviewed. Cross verification of data was done through telephonic & photographic information. Measures, which are taken to minimize sampling bias, are simple random sampling and a second reviewer to evaluate the sample size, which was selected, was 560. Data was collected from the database of Saveetha dental college. Patients who reported in the Department of Prosthodontics were selected for the study. Google sheet tabulation and SPSS importing of the data was done. Descriptive statistics tests were performed. Software used - SPSS version 26 was used. Independent variable such as race and time; Dependent variable being Age, sex and socioeconomic status are assessed. Chi Square test was used to evaluate the data.

## Results:

According to Anon and Ross classification scalloped and thin gingival biotype was present in 62.1 % patients. And flat and thick was present in 37.9% individuals.There is more prevalence of good pink aesthetic score[81.90%] in comparison to average pink aesthetic score[18.10%].There is a statistically significant association of gingival biotype with age and gender [p<0.05] (Table 1 - see PDF). There is no statistical significance of Anon and Ross classification with pink aesthetic score [p>0.05] (Table 2 - see PDF).

## Discussion:

In all age groups scalloped and thin gingival biotype was more common.In all age group thick biotype was more common.Females had scalloped and thin gingival biotypes more as compared to males,Females had thin gingival biotype more than males.Pink esthetic score didn't give any significant value in single crown cases 85% cases had a good pink aesthetic score. The dimensions of gingiva and different parts of the masticatory mucosa demonstrate considerable site and subject variability. They have become the subject of considerable interest in restorative and periodontics from both an epidemiologic, as well as a therapeutic point of view [12]. Various methods were proposed to measure gingival tissue thickness. There are various direct methods of measurement which include, TRAN, ultrasonic devices, and CBCT. In the direct method [12] the tissue thickness was measured using a periodontal probe. When the thickness was near or exact 1.5 mm, it was categorized as a thick biotype. With a thickness less than1.5 mm, it was considered a thin tissue biotype. Many factors have to be considered before probing that is the angulation and distortion of the tissue during probing.However, this method of measurement had several inherent limitations, such as the precision of the probe, which is to the nearest 0.5 mm, the angulation of the probe during the transgingival probing, and the distortion of the tissue during probing. Kan et al. [18] introduced the clinical assessment of gingival biotypes using a periodontal probe as an easy and low-cost method to evaluate gingival biotype. Eghbali and co-workers [19] in their study, showed the difficulties in correct visual assessment of the gingival biotype independent of the examiners experience. In the TRAN technique, the gingival biotype was labelled as thin when the part of periodontal probe showed through the gingival margin from inside the sulcus. The biotype was considered thick when the probe did not show through the gingival margin.Although this method was good and clinically reproducible, it had several drawbacks. Most importantly, drawbacks include difficulties in maintaining the directionality of the transducer, unavailability of the device, and high costs. Cone-beam computed tomography scans were used to visualize and measure the thickness of both hard and soft tissues [20] Limitations of this report might be that, e.g., previous orthodontic treatment was not considered as a factor possibly influencing soft tissue thickness. Sample size of the study can be increased and them maybe better results can be obtained.In my opinion according to the literature about application of the PES index to aesthetic evaluation of implant-prosthetic rehabilitation of the anterior sector, we also verified the validity of such index for full veneer prosthetic rehabilitation of the anterior area. The rightness of the PES index for the objective outcome assessment of the esthetic dimension of anterior single-tooth crown was confirmed. However, prospective clinical trials are needed to further validate and refine this index and its clinical use also for anterior full veneer single crown prosthetic rehabilitation.

## Conclusion:

Females have overall thin gingival biotype compare to males and in cases of single crown PES score is not very significant as soft tissue changes are very less.However, to the best of our knowledge, there is a paucity of evidence comparing the accuracy of these techniques used to ascertain tissue thickness. Most frequently used assessment methods for classifying the gingival biotype are not reliable and lack inter-examiner reproducibility. There is a clear need to define new diagnostic criteria and to develop more reliable assessment systems.
